# Albumin-Based Liver Reserve Models vs. MELD 3.0 in Prognostic Prediction for Hepatocellular Carcinoma Patients with Renal Insufficiency

**DOI:** 10.3390/ijms242316987

**Published:** 2023-11-30

**Authors:** Shu-Yein Ho, Po-Hong Liu, Chia-Yang Hsu, Hung-Ting Tseng, Yi-Hsiang Huang, Chien-Wei Su, Ming-Chih Hou, Teh-Ia Huo

**Affiliations:** 1 Division of Gastroenterology and Hepatology, Min-Sheng General Hospital, Taoyuan 33044, Taiwan; 2 School of Medicine, National Yang Ming Chiao Tung University, Taipei 11221, Taiwan; 3 Department of Internal Medicine, University of Texas Southwestern Medical Center, Dallas, TX 75390, USA; 4 VA Sierra Nevada Health Care System, Reno, NV 89502, USA; 5 Department of Medicine, Taipei Veterans General Hospital, Taipei 11217, Taiwan; 6 Healthcare & Services Center, Taipei Veterans General Hospital, Taipei 11217, Taiwan; 7 Institute of Clinical Medicine, National Yang Ming Chiao Tung University, Taipei 11221, Taiwan; 8 Department of Medical Research, Taipei Veterans General Hospital, Taipei 11217, Taiwan; 9 Institute of Pharmacology, National Yang Ming Chiao Tung University, Taipei 11221, Taiwan

**Keywords:** hepatocellular carcinoma, ALBI, PALBI, EZ-ALBI, PAL, MELD

## Abstract

The severity of liver functional reserve is an important prognostic predictor in hepatocellular carcinoma (HCC). The albumin–bilirubin (ALBI), easy (EZ)-ALBI, platelet-albumin–bilirubin (PALBI), platelet–albumin (PAL) score, and MELD 3.0 score are used to evaluate the severity of liver dysfunction. However, their prognostic role in HCC patients, specifically with renal insufficiency (RI), is unclear. We aimed to investigate the predictive accuracy of the five models in these patients. A total of 1120 newly diagnosed HCC patients with RI were enrolled. A multivariate Cox proportional analysis was used to identify independent predictors associated with survival. In the Cox model, older age, an α-fetoprotein ≥20 ng/mL, vascular invasion, a medium and high tumor burden score, poor performance status, a higher ALBI grade, an EZ-ALBI grade, a PALBI grade, a PAL grade, and MELD 3.0 score were all independently associated with decreased overall survival (all *p* < 0.001). Among the five liver reserve models, the ALBI grade is the best surrogate marker to represent liver functional reserve in terms of outcome prediction. The albumin-based liver reserve models (ALBI, EZ-ALBI, PALBI, and PAL) and MELD 3.0 are all feasible prognostic markers to indicate liver injury, specifically in HCC patients with RI. Among them, the ALBI grade is the most robust tool for survival prediction in these patients.

## 1. Introduction

Hepatocellular carcinoma (HCC) accounts for up to 90% of primary hepatic malignancies and is the fourth leading cause of cancer-related deaths worldwide [1]. The prognosis of HCC is usually dismal because the majority of patients are diagnosed at an advanced stage. Major risk factors for HCC are chronic viral hepatitis B and C, alcoholism, and non-alcoholic fatty liver disease (NAFLD) [2,3,4]. Available curative treatments, such as partial hepatectomy, transplantation, and local ablation therapy, are reserved for patients with early-stage HCC [5,6]. Alternatively, transarterial chemoembolization (TACE) and targeted or immunotherapy are mainly indicated for patients at intermediate or advanced cancer stages [7,8,9,10].

HCC typically develops from chronic liver disease or cirrhosis. Notably, liver cirrhosis is a predisposing factor for renal insufficiency (RI). The mechanism is related to decreased systemic vascular resistance, splanchnic arterial vasodilatation, and renin–angiotensin system activation. Notably, renal arterial vasoconstriction and hypoperfusion often further induce renal dysfunction [11]. RI, defined as an estimated glomerular filtration rate (eGFR) below 60 mL/min/1.73 m^2^, had a profoundly negative survival impact on HCC [12,13].

The liver functional reserve plays an important role in the management and prognostic prediction of HCC. The Child–Turcotte–Pugh (CTP) classification is traditionally used to assess liver dysfunction, but it has the drawbacks of subjective variables and inter-related variables [14]. The albumin–bilirubin (ALBI) grade, based only on serum albumin and bilirubin levels, is another tool to assess liver reserve. The ALBI grade can objectively assess liver reserve, and several independent study groups have validated its prognostic accuracy in different clinical scenarios [15,16,17,18,19]. The easy-ALBI (EZ-ALBI) grade, an updated version of the ALBI grade, is also a feasible model to indicate a liver injury and is highly correlated with the ALBI score. The EZ-ALBI grade demonstrated good prognostic discrimination across different HCC cancer stages and treatments [20,21,22]. A major step forward is to take a platelet count, a marker of portal hypertension in cirrhosis, into consideration, and the platelet–albumin–bilirubin (PALBI) grade was proposed in this regard. The PALBI grade incorporates the serum platelet count, albumin, and bilirubin levels to indicate liver reserve in HCC. The PALBI grade also displayed good prognostic accuracy in HCC patients undergoing surgical resection, radiofrequency ablation, TACE, and sorafenib treatment [23,24,25]. Furthermore, the platelet–albumin (PAL) grade, based only on the serum platelet and albumin levels, was also applied to assess liver reserve in HCC. The PAL grade could well discriminate long-term survival in HCC patients undergoing surgical resection and TACE [26,27,28].

More recently, the model for end-stage liver disease (MELD) score of 3.0, an updated version of the original MELD, incorporates serum albumin, bilirubin, creatinine, sodium level, and prothrombin time prolongation and has been used to evaluate liver reserve and prioritize the waiting list in liver transplantation candidates [29,30,31]. Given all these different models, selecting the prognostic tool in HCC becomes elusive. Our study was based on a large patient cohort to provide crucial prognostic information specifically on HCC patients with RI. This study aimed to comprehensively compare the predictive accuracy between the four albumin-based liver reserve models (ALBI, EZ-ALBI, PALBI, and PAL) and MELD 3.0 in these patients.

## 2. Results

### 2.1. Baseline Characteristics

The baseline demographics of HCC patients with RI are summarized in Table 1. Their mean age was 72 years, and most patients were male. Chronic hepatitis B and C were the main etiology of HCC. About 64% of patients had a single tumor, and the mean tumor diameter was 5.2 cm. Most patients had low tumor burden scores (TBS; 28%) and medium TBS (64%). Vascular invasion was present in 23% of patients, and 20% of patients had ascites formation. About 67% of patients had CTP classification A. The distribution of the ALBI grade, the EZ-ALBI grade, the PALBI grade, the PAL grade, the MELD 3.0 score, and treatment modalities are also described in Table 1. HCC patients with RI at a very early or early stage had longer overall survival compared with those with intermediate or advanced stages (*p* < 0.001). About 90% of early-stage HCC patients who had RI had received surgical resection or local ablation, and their 3- and 5-year survival rates were 67% and 54%, respectively.

### 2.2. Correlation between MELD 3.0 Score and ALBI, PALBI, PAL, and EZ-ALBI Scores

**Figure 1:**
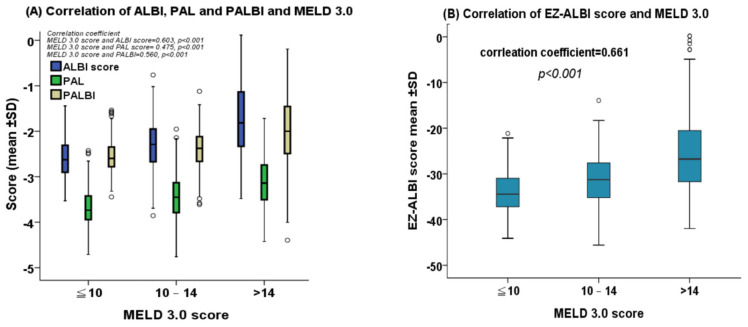
(**A**) The correlation between albumin–bilirubin (ALBI), platelet-albumin-bilirubin (PALBI), and platelet-albumin (PAL) scores versus the MELD 3.0 score. Patients with higher MELD 3.0 scores consistently had higher ALBI, PALBI, and PAL scores (all *p* < 0.001). (**B**) The correlation between the easy (EZ)-ALBI score and the MELD 3.0 score. Patients with higher MELD 3.0 scores had higher EZ-ALBI scores (*p* < 0.001). Each circle on the correlation figure represents one individual from the data set.

Patients with a higher MELD 3.0 score consistently had higher ALBI, PALBI, PAL, and EZ-ALBI scores. The correlation coefficient of the MELD 3.0 score vs. the ALBI, PAL, PALBI, and EZ-ALBI scores were 0.603, 0.475, 0.560, and 0.661, respectively (all *p* < 0.001; Figure 1A,B).

### 2.3. Kaplan–Meier Survival Analysis

**Figure 2:**
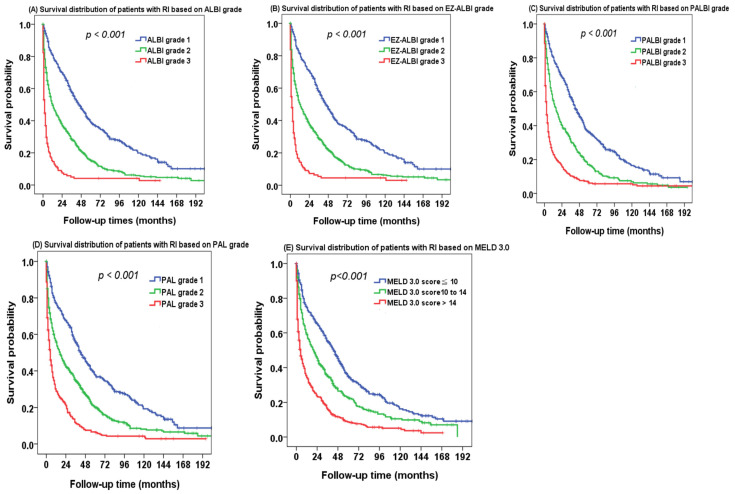
The survival distribution of hepatocellular carcinoma (HCC) patients with renal insufficiency (RI) based on (**A**) ALBI grade, (**B**) EZ-ALBI grade, (**C**) PALBI grade, (**D**) PAL grade, and (**E**) MELD 3.0 score. There was a significant survival difference in different ALBI, EZ-ALBI, PALBI, PAL grade, and MELD 3.0 scores (all *p* < 0.001).

There was a significant survival difference in different ALBI grades of HCC patients with RI (Figure 2A). The 1-, 3-, and 5-year overall survival rates were 73%, 59%, and 48% for ALBI grade 1, 46%, 30%, and 21% for ALBI grade 2, and 16%, 11%, and 9% for ALBI grade 3 patients, respectively. EZ-ALBI grade 1 patients also had better long-term survival compared with EZ-ALBI grade 2 and grade 3 patients (Figure 2B, *p* < 0.001). PALBI grade 3 patients consistently had poor survival compared with PALBI grade 1 and grade 2 patients (Figure 2C, *p* < 0.001). The PAL grade can likewise discriminate different overall survival in the study patients (Figure 2D, *p* < 0.001). Patients with higher MELD 3.0 scores had decreased survival compared with those with lower MELD 3.0 scores (Figure 2E, *p* < 0.001). The 1-, 3-, and 5-year survival rates were 64%, 49%, and 38% for a MELD 3.0 score < 10, 41%, 26%, and 18% for a MELD 3.0 score 10 to 14, and 23%, 13%, and 9% for a MELD 3.0 score > 14, respectively.

### 2.4. Multivariate Cox Proportional Hazards Model

In the Cox multivariate model (Table 2), age > 72 years (hazard ratio [HR]: 1.264, 95% confidence interval [CI]: 1.104–1.446, *p* < 0.001), AFP ≥ 20 ng/mL (HR: 1.856, 95% CI: 1.702–2.025, *p* < 0.001), vascular invasion (HR: 1.957, 95% CI: 1.643–2.332, *p* < 0.001), ascites (HR: 1.65, 95% CI: 1.406–1.936, *p* < 0.001), medium TBS (HR: 1.576, 95% CI: 1.341–1.852, *p* < 0.001), high TBS (HR: 2.076, 95% CI: 1.558–2.767, *p* < 0.001), PS 1–2 (HR: 1.304, 95% CI: 1.077–1.578, *p* < 0.001), and PS 3–4 (HR: 1.541, 95% CI: 1.221–1.726, *p* < 0.001) were independently associated with a decreased survival.

When the ALBI grade was included in the multivariate analysis (Cox model 1), ALBI grade 2 (HR: 1.432, 95% CI: 1.221–1.68, *p* < 0.001) and grade 3 (HR: 2.362, 95% CI: 1.852–3.013, *p* < 0.001) patients had an increased risk of mortality compared with ALBI grade 1 patients. Using a similar approach (Cox model 2), EZ-ALBI grade 2 (HR: 1.491, 95% CI: 1.273–1.747, *p* < 0.001) and grade 3 (HR: 1.758, 95% CI: 1.758–2.894, *p* < 0.001) predicted increased risk of death compared with EZ-ALBI grade 1. In Cox model 3, PALBI grade 2 (HR: 1.427, 95% CI: 1.491–1.747, *p* < 0.001) and grade 3 (HR: 2.255, 95% CI: 1.758–2.894, *p* < 0.001) were consistently associated with a shortened survival compared with PALBI grade 1. In Cox model 4, PAL grade 2 (HR: 1.381, 95% CI: 1.170–1.629, *p* < 0.001) and grade 3 (HR: 1.812, 95% CI: 1.503–2.184, *p* < 0.001) were also associated with increased mortality compared with PAL grade 1. Lastly, in Cox model 5, a MELD 3.0 score between 10 and 14 (HR: 1.220, 95% CI: 1.029–1.446, *p* = 0.022) and a MELD 3.0 score > 14 (HR: 1.851, 95% CI: 1.560–2.197, *p* < 0.001) independently predicted a decreased survival rate compared with the MELD score ≤ 10 group.

### 2.5. Comparison of Prognostic Performance

The comparison of the prognostic performance among ALBI, EZ-ALBI, PALBI, PAL, and MELD 3.0 scores showed that the ALBI score had the lowest corrected Akaike information criteria (AICc) and highest homogeneity value, followed by the PALBI score and the EZ-ALBI score (Table 3); these data indicate that the ALBI score is the best model to evaluate the severity of liver dysfunction specifically in HCC patients with RI.

## 3. Discussion

Patients with HCC often have co-existing liver cirrhosis that may, in turn, predispose them to RI. Previous studies showed that up to 25% of HCC patients had variable degrees of RI [11,13]. The four albumin-based liver reserve models (ALBI, EZ-ALBI, PALBI, and PAL) are widely used to evaluate liver reserve in patients with HCC. Alternatively, MELD 3.0 has been proposed as a surrogate marker to indicate liver reserve in liver transplant candidates. However, these different models have not been specifically assessed in HCC patients with RI. Our results are based on a large patient cohort that may provide sufficient information. This study, which comprised a large patient cohort, shows that all five models are feasible methods to indicate liver reserve in this special clinical setting. Notably, among these models, the ALBI grade is the best marker to represent the liver functional reserve regarding outcome prediction.

HCC typically develops from the background of chronic liver disease or liver cirrhosis. The serum albumin level is well known for its predictive ability in patients with liver cirrhosis. Hypoalbuminemia is often associated with advanced cirrhosis and exerts an adverse impact on the clinical outcome of cirrhotic patients [31]. Although the traditional CTP score has been used in HCC for decades, it has inevitable drawbacks. The ALBI score is a simple score derived from an easily accessible and objective marker used to examine liver reserve in HCC patients. Previous studies have reported the reliability of the ALBI grade in prognostic stratification for HCC patients [19]. The ALBI was validated by several independent study groups, showing good prognostic power and the ability to define patient management. Consistently [15,16,17,18,19], we demonstrated that the ALBI grade can well discriminate survival in HCC patients with RI. Alternatively, the EZ-ALBI score is an easy and user-friendly tool to indicate liver functional reserve in HCC. The EZ-ALBI score has been validated in a previous study showing good discriminatory ability for the long-term survival of HCC patients across different cancer stages and treatments [22]. Other studies reported that EZ-ALBI grade 1 was associated with better outcomes than EZ-ALBI grades 2 and 3, suggesting that the EZ-ALBI grade is a feasible model to stratify patient outcomes in this setting [20,21,22].

Not considered in the ALBI and EZ-ALBI scores, platelet count is an indispensable surrogate marker for portal hypertension in cirrhosis. The PALBI grade, which includes the platelet count, albumin, and bilirubin levels, was introduced to assess the severity of liver injury and can adequately stratify patient outcomes. In this study, PALBI grades 2 and 3 predicted decreased survival compared with PALBI grade 1. The results were consistent with previous studies, and the PALBI grade is a useful tool for prognostic prediction in HCC patients with RI [24,25,32,33,34]. The PAL grade is also a new model to assess liver dysfunction in HCC patients undergoing surgical resection and TACE patients. Consistent with previous studies [26,27,28], we found that PAL grades 2 and 3 were associated with an increased mortality risk compared with PAL grades 1, indicating that the PAL is a feasible prognostic tool to stratify patient outcomes. Taken together, all four albumin-based models (ALBI, EZ-ALBI, PALBI, and PAL) can discriminate patient survival [15,16,17,18,19,20,21,22,23,24,25,26,27]. Therefore, these models are helpful in patient selection and guiding treatment allocation. Alternatively, MELD 3.0, in which serum albumin is considered, can also stratify different survival rates in these patients. Our data suggest that the serum albumin level is important in outcome prediction in HCC patients with RI.

Although the four albumin-based models and MELD 3.0 are feasible survival predictors, the performance of their prognostic accuracy is uncertain. We further demonstrate that among these five models, the ALBI grade had the lowest AICc and highest homogeneity value, indicating that the ALBI grade is a very robust model for evaluating liver reserve in these patients. As such, the ALBI grade should be used as a prognostic marker in cancer staging and treatment planning in future clinical trials.

HCC patients with RI at very early or early stages had longer overall survival compared with intermediated or advanced stages (*p* < 0.001). About 90% of HCC patients with RI in the early stages have received surgical resection and local ablation treatments.

However, there are some limitations of this study. Firstly, the study was performed in a single medical center in Taiwan with a high prevalence of HBV infection, which may limit the generalizability of the findings to other populations. Secondly, the cause of renal insufficiency was not specifically determined in the study, which may hinder the applicability of the findings to all study subjects. Thirdly, since MELD 3.0 is a continuous variable, whether a certain cutoff value could be appropriately used for optimal decision making is unknown.

## 4. Materials and Methods

### 4.1. Patients

A total of 1120 newly diagnosed HCC patients with RI were prospectively identified and retrospectively analyzed between 2002 and 2018. Their baseline characteristics such as age, sex, etiology of liver disease, serum laboratory data, tumor burden, severity of liver dysfunction, tumor staging, and treatment were recorded at the time of diagnosis. The individual patient’s treatment was discussed at the multidisciplinary HCC board of Taipei Veterans General Hospital. After treatment, patients were followed up every 3 months until drop-out or death. This study complies with the standards of the Declaration of Helsinki and current ethical guidelines and has been approved by the Institutional Review Board of Taipei Veterans General Hospital.

### 4.2. Diagnosis and Definition

The diagnosis of HCC was conducted according to current European and American HCC practice guidelines [5,35]. The eGFR was calculated using the modification of diet in renal disease (MDRD) equation: eGFR (mL/min/1.73 m^2^) = 186 × (creatinine (mg/dL))^−1.154^ × (age (years))^−0.203^ × 0.742 (if female). All patients in this study were ethnically Chinese. RI was defined as an eGFR below 60 mL/min/1.73 m^2^ [36]. The serum creatinine level used in the MDRD formula was the level recorded at the time of diagnosis.

The calculation of the tumor burden score (TBS) is as follows: TBS^2^ = (maximum tumor diameter)^2^ + (number of tumors)^2^. Patients were stratified accordingly into three groups: high TBS (over 13.74), medium TBS (3.36–13.74), and low TBS (less than 3.36) [37]. Performance status was evaluated using the Eastern Cooperative Oncology Group [38]. Vascular invasion was defined as radiological evidence of tumor invasion to intrahepatic vasculatures, portal trunk, or abdominal great vessels [39]. The definition of the formula and grading of ALBI, EZ-ALBI, PAL, PALBI, and MELD 3.0 are described in Table 4 [15,20,27,29].

### 4.3. Statistics

The comparison of categorical variables between the two groups was analyzed using the two-tailed Fisher exact test and Chi-squared test. The Mann–Whitney U test was applied to compare the continuous variables. The Pearson correlation method was used to estimate the relationship between the four albumin-based liver reserve models and MELD 3.0. The survival analysis was analyzed using the Kaplan–Meier method with a log-rank test. Independent prognostic predictors were determined using the multivariate Cox proportional hazards model. The prognostic performance among different liver reserve models was compared using the corrected Akaike information criteria (AICc) and homogeneity. The lower the AIC, the more explanatory and informative the model is. A *p*-value less than 0.05 was considered statistically significant. All statistical analyses were conducted using IBM SPSS Statistics for Windows, Version 21.0 (IBM Corp., Armonk, NY, USA).

## 5. Conclusions

This study was based on a large patient cohort to provide crucial prognostic information on HCC patients with RI. Our results indicate that albumin-based liver reserve models (ALBI, EZ-ALBI, PALBI, and PAL) and MELD 3.0 are all feasible prognostic models for HCC patients with RI. Among them, the ALBI grade is the best for outcome prediction and should be used as a surrogate marker in cancer staging and treatment planning. These findings may help contribute to fine-tuning patient management and prognostic evaluation. Further study is required for confirmation.

## Figures and Tables

**Table 1:** Baseline characteristics of hepatocellular carcinoma patients with renal insufficiency.

Variable	n = 1120
Age (years, mean ± SD)	72 ± 11
Male/female, n (%)	816/304 (73/27)
Etiologies of liver disease	
HBV, n (%)	403 (36)
HCV, n (%)	310 (27)
HBV + HCV, (%)	49 (4)
Others, (%)	358 (32)
Laboratory values (mean ± SD)	
Albumin (g/L)	3.5 ± 0.6
Bilirubin (mg/dL)	1.7 ± 3.9
Creatinine (mg/dL)	2.0 ± 1.7
Sodium (mmol/L)	138 ± 5
INR of PT	1.1 ± 0.5
Platelet (1000 ul/L)	174 ± 95
GFR (mL/min/1.73 m2)	43 ± 15
Diabetes mellitus, n (%)	430 (38)
AFP (ng/mL), median [IQR]	30 [5–861]
Tumor nodules (single/multiple), n (%)	712/408 (64/36)
Tumor size, mean ± SD	6.2 ± 4.5
Tumor size > 3 cm, n (%)	681 (68)
Tumor burden score, median, [IQR]	5.3 [3.2–9.7]
Tumor burden score (low/medium/high), n (%)	310/718/92 (28/64/8)
Vascular invasion, n (%)	255 (23)
Ascites, n (%)	313 (28)
CTP score (mean ± SD)	6.4 ± 1.7
CTP class (A/B/C), n (%)	749/288/83 (67/26/8)
ALBI score(mean ± SD)	−2.21 ± 0.71
ALBI grade (1/2/3)	372/601/147 (33/54/13)
EZ-ALBI score (mean ± SD)	−30.05 ± 8.06
EZ-ALBI grade (1/2/3)	346/639/135 (31/57/12)
PALBI score (mean ± SD)	−2.30 ± 0.56
PALBI grade (1/2/3)	452/323/345 (40/29/31)
PAL score (mean ± SD)	−3.41 ± 0.55
PAL grade (1/2/3)	304/560/256 (27/50/23)
MELD 3.0 score (mean ± SD)	14.64 ± 6.2
MELD 3.0 (≤10/10–14/>14)	393/318/409 (35/28/37)
Performance status (0/1/2/3–4)	566/174/203/177 (51/15/18/10/16)
BCLC (0/A/B/C/D) n (%)	66/241/177/436/200 (6/22/15/38/19)
Treatment	
Surgical resection	209 (19)
Liver transplantation	6 (1)
Percutaneous ablation	226 (20)
TACE	329 (29)
Others	350 (31)

Abbreviations: ALBI, albumin–bilirubin; AFP, α-fetoprotein; CTP, Child–Turcotte–Pugh; EZ-ALBI, easy albumin–bilirubin; INR of PT, international normalized ration of prothrombin time; MELD, model of end-stage liver disease; HBV, hepatitis B virus; HCV, hepatitis C virus; PALBI, platelet-albumin–bilirubin; PAL, platelet–albumin; TACE, transarterial chemoembolization; SD, standard deviation.

**Table 2:** Multivariate analysis of HCC patients with renal insufficiency (n = 1120).

	Univariate Analysis			Multivariate Analysis		
Overall Survival	HR	CI	*p*	HR	CI	*p*
Age (≤72/>72 years)	1.269	1.112–1.449	<0.001	1.264	1.104–1.446	<0.001
Sex (male/female)	0.997	0.908–1.052	0.543			
HBsAg (negative/positive)	1.222	1.069–1.396	0.003			
Anti-HCV (negative/positive)	0.945	0.822–1.085	0.421			
Albumin level (≥3.5/<3.5 g/dL)	2.221	1.947–2.535	<0.001			
Bilirubin level (≤1.7/>1.7/mg/dL)	2.275	1.925–2.690	<0.001			
Platelet (≥150,000/<150,000/μL)	1.195	1.104–1.295	<0.001			
INR of PT (<1.1/≥1.1)	1.143	1.005–1.301	0.042			
AFP level (<20/≥20 ng/mL)	1.919	1.680–2.191	<0.001	1.856	1.702–2.025	<0.001
Multiple tumors (no/yes)	1.456	1.275–1.662	<0.001			
Tumor size (≤3 cm/>3 cm)	2.161	1.867–2.500	<0.001			
Vascular invasion (no/yes)	3.481	2.983–4.063	<0.001	1.957	1.643–2.332	<0.001
Ascites (no/yes)	2.664	2.231–3.072	<0.001	1.650	1.406–1.936	<0.001
Tumor burden score						
Low	1			1		
Medium	1.997	1.710–2.333	<0.001	1.576	1.341–1.852	<0.001
High	5.683	4.391–7.355	<0.001	2.076	1.558–2.767	<0.001
Performance status						
0	1			1		
1–2	1.800	1.496–2.166	<0.001	1.304	1.077–1.578	<0.001
3–4	2.709	2.337–3.139	<0.001	1.541	1.221–1.726	<0.001
**Model 1**						
ALBI						
grade 1	1			1		
grade 2	2.051	1.766–2.382	<0.001	1.432	1.221–1.680	<0.001
grade 3	5.057	4.082–6.265	<0.001	2.362	1.852–3.013	<0.001
**Model 2**						
EZ-ALBI						
grade 1	1			1		
grade 2	2.010	1.727–2.339	<0.001	1.491	1.273–1.747	<0.001
grade 3	4.805	3.846–6.002	<0.001	2.255	1.758–2.894	<0.001
**Model 3**						
PALBI						
grade 1	1			1		
grade 2	1.755	1.495–2.061	<0.001	1.427	1.211–1.682	<0.001
grade 3	3.465	2.947–4.051	<0.001	1.812	1.503–2.184	<0.001
**Model 4**						
PAL						
grade 1	1			1		
grade 2	1.681	1.430–1.975	<0.001	1.381	1.170–1.629	<0.001
grade 3	3.092	2.564–3.730	<0.001	1.827	1.490–2.422	<0.001
**Model 5**						
MELD 3.0 score						
≤10	1			1		
10–14	1.473	1.246–1.742	<0.001	1.220	1.029–1.446	0.022
>14	2.692	2.300–3.150	<0.001	1.851	1.560–2.197	<0.001

Abbreviations: AFP, alpha-fetoprotein; ALBI, albumin–bilirubin; ALT, alanine aminotransferase; anti-HCV, hepatitis C virus antibody; eGFR, estimated glomerular filtration rate; EZ-ALBI, easy albumin–bilirubin; HBsAg, hepatitis B virus surface antigen; MELD, model for end-stage liver disease; INR of PT, international normalized ration of prothrombin time; PAL, platelet–albumin; PALBI, platelet–albumin–bilirubin.

**Table 3:** Comparison of the prognostic accuracy for albumin-based liver reserve models vs. MELD 3.0 in hepatocellular carcinoma patients with renal insufficiency (n = 1120).

Liver Reserve Models	Homogeneity (Wald χ^2^)	AICc
ALBI	239.031	11,281.79
EZ-ALBI	203.652	11,316.459
PALBI	221.884	11,298.227
PAL	136.085	11,384.026
MELD 3.0	156.026	11,364.088

**Table 4:** Formula and grading of non-invasive liver reserve models.

Non-Invasive Liver Reserve Models	Formula
ALBI, grade 1/2/3 (≤−2.6/>−2.6 and ≤−1.39/>−1.39)	(log(Bilirubin [μmol/L]) × 0.66) + (Albumin [g/L] × −0.085)
EZ-ALBI, grade 1/2/3 (≤−34.4, >−34.4 and ≤−22.2, >−22.2)	T.Bil (mg/dL) − (9 × Alb (g/dL))
PALBI grade 1/2/3/(≤−2.53), score>−2.53 and ≤−2.09)/(score>−2.09).	2.02 × log_10_bilirubin [μmol/L] level − 0.37 × (log_10_bilirubin level)^2^ − 0.04 × albumin level − 3.48 × log_10_platelet count [1000/μL] (PLT) + 1.01 × (log_10_PLT)^2^
PAL grade 1/2/3 ≤−3.77, >−3.77 and ≤−3.04, >−3.04	0.777 × albumin [g/dL] − 0.575 × log_10_ (platelet count) [10^4^/μL].
MELD 3.0 * (≤10, score 10, between and l4, >14)	1.33 (if female) + [4.56 × log_e_(bilirubin (mg/dl))] + [0.82 × (137 − Na (mEq/L))] − [0.24 × (137 − Na) × log_e_(bilirubin)] + [9.09 × log_e_(INR)] + [11.14 × log_e_(creatinine)] − [1.85 × (3.5 − albumin(g/dL))] − [1.83 × (3.5 −albumin) × log_e_(creatinine)] + 6

ALBI, albumin–bilirubin; EZ-ALBI, easy albumin–bilirubin; Na, sodium; PALBI, platelet–albumin–bilirubin; PAL, platelet–albumin; MELD, model for end-stage liver disease. * Serum creatinine level greater than 3.0 mg/dL was set as 3.0 mg/dL. Serum bilirubin, INR, and creatinine levels below 1.0 were set at 1.0 mg/dL. The lower and upper bounds of sodium were 125 mEq/L and 137 mEq/L, and the lower and upper bounds of albumin were 1.5 g/dL and 3.5 g/dL, respectively.

## Data Availability

Data are contained within the article.
